# Assessment of Two School-Based Programs to Prevent Universal Eating Disorders: Media Literacy and Theatre-Based Methodology in Spanish Adolescent Boys and Girls

**DOI:** 10.1155/2015/328753

**Published:** 2015-02-23

**Authors:** Marisol Mora, Eva Penelo, Teresa Gutiérrez, Paola Espinoza, Marcela L. González, Rosa M. Raich

**Affiliations:** ^1^Unitat d'Avaluació i Intervenció en Imatge Corporal, Departament de Psicologia Clínica i de la Salut, Universitat Autònoma de Barcelona, Bellaterra, 08193 Barcelona, Spain; ^2^Laboratori d'Estadística Aplicada, Departament de Psicobiologia i Metodologia de les Ciències de la Salut, Universitat Autònoma de Barcelona, Bellaterra, 08193 Barcelona, Spain

## Abstract

*Aims*. To evaluate the long-term effects of two school-based prevention programs administered to a universal mixed-sex sample of school-going adolescents on disturbed eating attitudes, aesthetic ideal internalization, and other eating disorder risk factors, when compared to a control group. *Methods*. Participants were 200 adolescents aged 12–15 selected by means of incidental sampling from second-year compulsory secondary education at schools. An interactive multimedia media literacy program (ML + NUT, *Media Literacy* and *Nutrition*) and a program focused on the same topics using dramatic arts (*Theatre Alive*) were applied and compared with a control group. Pretest, posttest (1 month later), and 5- and 13-month follow-up measurements were taken. Analyses were conducted with two-way mixed 3 × 3 ANCOVA (group × phase) adjusted by baseline levels, body mass index, and sex. *Results*. Participants in both experimental groups showed significantly higher self-esteem scores than the control group over time. The ML + NUT group also presented lower aesthetic ideal internalization scores than the control group. *Discussion*. Both programs can benefit students' self-esteem. Moreover, ML + NUT program was useful in reducing thin-ideal internalization. However, differences in body dissatisfaction and disordered eating attitudes were not found. The programs may be protective on the core psychological variables, which are essential to adaptive adolescent development.

## 1. Introduction

In different countries of Europe, in community samples, eating-related problems in around 15–30% of adolescent girls have been reported [1]. These rates are a preoccupying public health issue among adolescent population. Additionally, the dismal prognosis and poor treatment outcomes for eating disorders (ED) advise preventive programs as an urgent endeavour. As suggested by Wilksch et al. [2], it is crucially important to design theoretically solid ED prevention programs targeting prospectively identified ED risk factors, such as perceived pressure to be thin, aesthetic ideal internalization, body dissatisfaction, restrictive dieting, negative affect, substance use [3, 4], and self-esteem [5]. Regarding pressure to be thin, the media have an important influence on the health, and more specifically on the eating behaviours, of young people of both sexes; media models' influence was the main predictor driving thinness in both males and females [6]. A meta-analysis of laboratory studies reviewing media effects based on experimental presentations of the ideal physique on ED symptoms shows that not only are women susceptible to the idealized images of beauty presented by the media, but men are also vulnerable [7].

Youth may spend 33–50% of their time with some form of mass media [8]. Instead of trying to protect youth from potentially detrimental messages and the health and wellbeing of students as a way of developing resilience to sociocultural messages, media literacy education involves them in a critical activity to examine media messages that influence their perceptions and practices [8]. Media literacy education aims to provide youth with the critical cognitive skills needed to neutralize the influence of those messages in their thinking and behaviour and to challenge the glorification of thinness for girls and the muscular ideal for boys. Several studies have found that media literacy interventions decreased thin-ideal internalization and other related risk variables [2, 7, 9]. The most common approach used by effective classroom-based preventive body image programs was media literacy, which was used by 86% of the programs [10].

Eating disorder prevention programs produced significant reductions in measures of eating disorder symptoms according to Stice et al.'s review [11]. Programs that were focused on reducing thin-ideal internalization significantly diminished the initial symptoms of ED and the risk of the onset of clinical or subclinical ED [4].

Bearing in mind this consideration, we developed a universal school-based disordered eating prevention program aimed at reducing disturbed eating attitudes and diminishing ideal aesthetic internalization. Thus, its components were designed to modify some ED risk factors identified prospectively [11, 12] based on Bandura's Social Cognitive model [13] and the Media Literacy perspective [14]. Our program has a multimedia and interactive format and is conducted at schools. The first version had two components: learning the basic concepts of nutrition (NUT) and criticism of the prevailing female aesthetic beauty model of extreme thinness and media literacy involving a critique of information presented by the mass media (ML). After preliminary studies [15–17], this program was expanded and improved, giving it a more interactive format, increasing the number of sessions and incorporating new activism and media literacy activities.

This modified intervention was evaluated by several studies [17–19] in adolescents of both sexes. Favourable findings on a decrease in internalisation of the thin ideal, disordered eating attitudes, and body dissatisfaction in boys and girls are encouraging, despite the fact that the ML component was initially only addressed to girls [18, 19], as most prevention programs are. There may be a learning procedure which leads boys to criticise the advertising targeted at females and also to generalise this process to advertising targeted at males [19]. Therefore, preventive programs aimed at both males and females should be implemented. With this goal in mind, an additional critical analysis section of the male aesthetic model and its treatment in the media was developed, along with a gender comparison session of both aesthetic models portrayed in the media, which are outlined elsewhere [20].

Recent studies have verified that theatre can play a preventive role in weight-related problems, since performing in a play offers adolescents a leadership opportunity in which they get to teach, thus increasing their sense of self-empowerment and ownership of the messages [21, 22]. Very few preventive disordered eating programs involve drama methods; nevertheless, drama approach can be a potential space for discussion, critical reflection, and self-improvement in an educational context. Hence, the next research step was to develop and implement a theatre-based program following the media literacy philosophy. Our preventive theatre program is based on one genre of theatre in education (TIE) called theatre in health education (THE), according to Joronen et al. [23]. It combines theatre techniques in order to promote health education. Drama has an interesting component underscored by these authors, as the learning process is derived from the relationship between fiction and reality, which can serve as a rehearsal for real life. Moreover, it is an excellent scenario to learn by observing and receiving feedback from others.

Hence, the purpose of this study is to evaluate the middle- and long-term effects of two school-based prevention programs administered to a universal mixed-sex sample of school-going adolescents on disturbed eating attitudes, aesthetic ideal internalization, and other ED risk factors such as body dissatisfaction and self-esteem. Thus, the interactive multimedia program based on the philosophy of media literacy and another program focused on the same topics but with a methodology based on the dramatic arts were both compared to a control group at posttreatment and at 5- and 13-month follow-ups. We hypothesized that over time both groups receiving preventive interventions would experience lower disturbed eating attitudes, aesthetic ideal internalization, and body dissatisfaction and higher self-esteem than the nontreatment control group.

## 2. Method

### 2.1. Participants

Participants were recruited from second-year compulsory secondary education at five urban state and state-subsidised schools in the city of Manresa, located in the Barcelona region (Catalonia, Spain), who agreed to participate. They were selected by means of incidental sampling. Of the 207 initial participants, data were obtained from 200 adolescents (96.6%; 100 girls and 100 boys) aged 12–15 (M = 13.4 years; SD = 0.5), the reduction in sample size being due to some of the questionnaires not being fully answered. Mean body mass index, based on in situ measurements of height and weight, was 20.0 (SD = 3.1) for girls and 20.6 (SD = 3.6) for boys. Weight status, according to international criteria that take sex and age into consideration [24, 25], was 4.0% underweight, 77.9% normal weight, and 13.6% overweight, with the remaining 4.5% classified as obese. Socioeconomic status based on parents' education and occupation [26] was as follows: 24.9% high, 41.8% medium and medium-high, and 33.3% medium-low and low. The distribution of participants in terms of origin was 79.0% from Spain/Europe, 12.0% from Morocco, 7.0% from Central and South America, and 2.0% from other countries.

**Figure 1:**
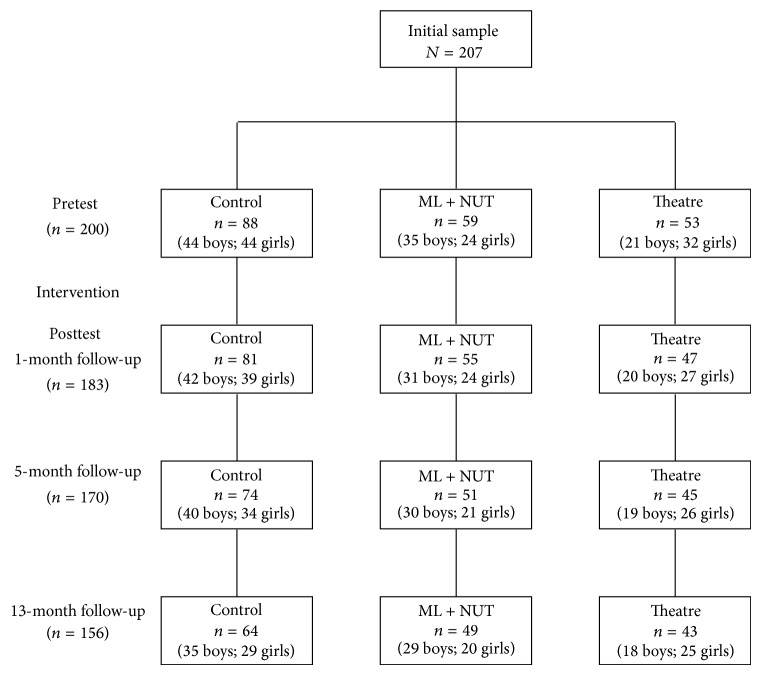
Flow diagram of progress through the phases of the study.

The schools were assigned to the experimental conditions taking into account the schools as the sample unit, not individual participants, in order to avoid the spill-over effect between the experimental condition participants. Therefore, the biggest school served as the control group, two schools received the ML + NUT program, and two more schools were assigned to the theatre program. One hundred and fifty-six participants (75.4%) remained until the last follow-up, with the reduction in sample size being due to absences on the testing days, changes in school, or blank questionnaires being returned (Figure 1).

### 2.2. Materials

#### 2.2.1. Sociobiographical Data

Personal, family, and social details were collected; we ascertained the socioeconomic status based on the parents' educational level and occupation according to Hollingshead's index [26].

#### 2.2.2. Weight and Height

Weight and height measurements were taken using a 100 g Tefal scale sensitive computer and a 2 m Kawe Kw 444440 human height measuring system, and then data were averaged and body mass index (BMI) was calculated.

#### 2.2.3. Eating Attitudes Test (EAT-26) [27]

This 26-item self-reported questionnaire assesses feelings and behaviours that are characteristic of individuals with disordered eating and is therefore considered a good screening tool to assess and identify people at risk of having an ED. The items provide six response options ranging from 1 (*never*) to 6 (*always*); the three least pathological responses receive 0 points and the other responses receive 1, 2, and 3 points to denote increasing severity. The total score is obtained by a sum of the items, after reversing them when necessary, and higher scores indicate more disordered eating. We applied the Spanish adaptation [28], which has adequate psychometric characteristics. In the present sample, the internal consistency for the total score was excellent (*α* ≥ .91 for each assessment point). The cutoff is a score of 10.

#### 2.2.4. Sick, Control, One, Fat, Food Questionnaire (SCOFF) [29]

The SCOFF is a screening questionnaire to assess disordered eating. It contains 5 dichotomous questions (*yes*/*no*), and the total score is the sum of the positive answers. We applied the Spanish adaptation [30] of this test. The internal consistency for the total scores of this short tool in the current sample was low (KR20 between .46 and .56 for each assessment point) but similar to that found in other studies in adolescents [31]. A score equal to or above 2 has been considered as a risk indicator.

#### 2.2.5. Sociocultural Attitudes towards Appearance Questionnaire-R (SATAQ-R) [32]

This self-reported questionnaire was developed to assess awareness of and the tendency to strive toward social standards of appearance [33]. We used the Spanish version [34] with both a male and a female version containing 21 items adapted for each sex, which provide five response options ranging from 1 (*strongly disagree*) to 5 (*strongly agree*). The SATAQ-R consists of two subscales: awareness (11 items) and internalization (10 items). Awareness has been defined as knowledge that a standard of appearance exists, while internalization is a profound incorporation or acceptance of these values that affects one's attitudes or personal behaviour. Scale scores are obtained by the average of the corresponding items, after reversing them when necessary, and higher scores indicate a stronger presence of the corresponding construct. In the present sample, the internal consistency was satisfactory for both the awareness and internalization scale scores (*α* ≥ .81 and *α* ≥ .82, resp., for each assessment point), similar to Cusumano and Thompson [32].

#### 2.2.6. Contour Drawing Rating Scale (CDRS) [35]

The CDRS consists of nine drawings of a female or male figure, depending on the sex of the respondent. Each drawing increases in size from* extremely thin* (1) to* very obese* (9). Participants are asked to rate their ideal figure (what they ideally want to look like) and their current size (perceived figure). We used the discrepancy between the ideal and current size scores in absolute value as an index of body size dissatisfaction, which can range from 0 to 8, with higher scores indicating more dissatisfaction. Evidence of satisfactory validity and test-retest reliability in male and female undergraduates and early adolescent girls has been reported [35, 36].

#### 2.2.7. Rosenberg Self-Esteem Scale (RSES) [37]

We used the Spanish adaptation [38]. This is a 10-item self-reported measure of overall self-esteem. The items provide four response options ranging from 1 (*strongly disagree*) to 4 (*strongly agree*). The total score is obtained by a sum of the items, after reversing them when necessary, and higher scores indicate better self-esteem. Satisfactory psychometric properties have been demonstrated for the English version [39], as well as for the Spanish version adapted for adolescents [40]. In the present sample, the internal consistency for the total score was satisfactory (*α* ≥ .81 for each assessment point).

Cronbach's alpha values for all questionnaire measures were equivalent across sex (*P* > .01), since *α* comparison tests showed no statistical differences between groups, except for the RSES scale score (.85 for girls and .72 for boys; *P* = .008).

### 2.3. Procedure

This study was approved by the Ethics Committee of our institution and was approved and mediated by the Manresa Municipal Institute of Health and Social Welfare. Informed written consent from parents and oral consent from adolescents were obtained. We applied a quasi-experimental design with randomisation at the school level, not for each participant individually, with prospective assessments at pretest, posttest (1 month after the intervention), first follow-up (5 months), and second follow-up (13 months).

While the control participants took normal classes, the participants assigned to the improved ML + NUT or theatre program participated in the interventions once a week in 10 sessions of 120 minutes each facilitated by some of the authors of the program and trained psychologists and professional actors. These sessions were held during academic year 2011-2012 in mixed-sex classes with an average of 30 students per class. Data were collected by our team at the schools, ensuring that there were two team members for each class plus the teacher in charge. After securing the teachers' agreement and after issuing general instructions in class, the participants answered the assessment battery individually and anonymously in the classroom during normal class time. Confidentiality was assured, as was the possibility of getting feedback through a mnemonic code. In situ height and weight measures were taken by two members of the team individually and privately to ensure confidentiality.

#### 2.3.1. Disordered Eating Prevention Program

“*Eating, feminine aesthetic beauty model, and the mass media: how to train critical students in secondary school*” [41]: the two main original components of the program targeted at the adolescent population, nutrition knowledge (NUT) and media literacy (ML), were updated. In the NUT component, which is aimed at correcting false beliefs on nutrition by providing knowledge about balanced eating, new activities were added, such as food self-recording and others, in order to encourage the practice of regular physical activity and reduce the time spent on sedentary activities. Likewise, a test of the five senses with four different foods was conducted. In the ML component, a new module aimed at criticising the current male aesthetic model and a session in which female and male models were compared were added in order to uncover the social construction of gender ideals and roles.

Furthermore,* activism activities* that involve critical analysis of advertisements were proposed, such as learning about how to write letters of complaint to the media, as well as new activities about a collective and collaborative production including both an “analysis of advertising messages” and a “parody making-of video” from fashion advertisements (Table 1).

#### 2.3.2. “*Theatre Alive*”

This program is based on the premises of social cognitive theory [13] and the ML approach. Through the theatre program, students develop the five communication competencies essential to literacy practices: accessing, analyzing, creating, reflecting, and acting [42]. Live drama may also be an effective way to engage parents in school-based interventions [21] and to communicate messages about the importance of parental influence and support for the desired behaviour change [43]. In this regard, parents can build inquiry and critical thinking skills about media messages as a complement to the work being done at school. The “*Theatre Alive*” program is an intervention that was designed to impact not only the individual level but also the group, family, and community (school) levels. “*Theatre Alive*” promotes critical analysis of the ideals of male and female beauty that are transmitted in the media, revealing the tricks and montages used in advertisements. The program is based on preparing and performing a play called “Teen Spirit.” A special script created by playwright J. Faura with advice from our research team was developed for the play using the core of the media literacy multimedia program along with information gathered in interviews with adolescents.

Students learned the script and dramatic expression in 10 sessions conducted during class time under the guidance of professional actors. Each student played a character in the play. The program ended with a performance for parents and the school community.

### 2.4. Statistical Analysis

The statistical analyses were carried out with SPSS19 [44]. Differences in baseline measures between participants who completed the three follow-ups (nondropouts) and participants who were absent (dropouts) were examined using *t*-test procedures. Next, ANCOVA procedures were used to assess the effectiveness of the ML + NUT and* Theatre Alive* programs over follow-ups, as in previous studies [18, 19, 45]. This involved a 3 (group: ML + NUT,* Theatre Alive* and control) × 3 (phase: postprogram 1 month after the intervention, 5-month follow-up, and 13-month follow-up) two-way mixed design adjusted by preprogram scores, BMI, and sex, for each of the six outcome variables considered: EAT-26, SCOFF, SATAQ-R (Internalisation and Awareness), CDRS, and RSES. For hierarchical models as ours, the interaction effect (group × phase) was evaluated first and, when not statistically significant, main effects were then interpreted, the *α* level being set at .05. Corrections for lack of homogeneity and/or lack of sphericity were applied when necessary, by adjusting the degrees of freedom of the *F*-distribution (lower-bound adjustment method for the former). When the main effect of group was found to be statistically significant in absence of a higher-order interaction effect, Bonferroni-adjusted post hoc analyses were conducted to value mean differences (95% confidence intervals) among groups, and effect sizes were calculated through Cohen's *d* ([46]; standardized difference between both means). For the latter, results were interpreted as small if *d* values were around 0.2, medium for *d* values around 0.5, and large for *d* values above 0.8 [47]. The listwise deletion method was used.

## 3. Results

### 3.1. Data Screening

Participants who completed the three follow-ups (nondropouts included in mixed analyses, *n* = 156) and participants who were absent (dropouts excluded through listwise deletion, *n* = 44) did not differ in terms of sex (*P* = .172), age (*P* = .658), group allocation (*P* = .223), weight status (*P* = .067), BMI (*P* = .348) or baseline SCOFF scores (*P* = .101), self-esteem (*P* = .089), SATAQ-R awareness (*P* = .974), and SATAQ-R internalisation (*P* = .491). However, the participants who remained scored lower at baseline than those who dropped out at any assessment point for EAT (M = 6.7, SD = 8.6 versus M = 10.1, SD = 10.2; *P* = .026) and CDRS body dissatisfaction (M = 0.5, SD = 1.2 versus M = 1.2, SD = 1.9; *P* = .024).

### 3.2. Comparisons among Groups over Time

**Figure 2:**
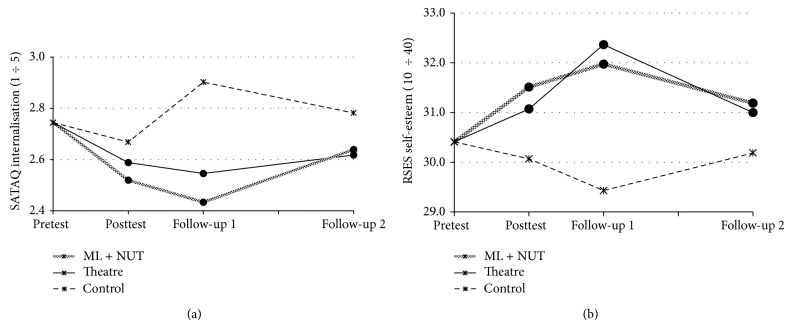
Profiles for statistically significant comparisons among groups for SATAQ-R internalization (a) and RSES self-esteem scores (b).

Table 2 (left) presents the observed means (and SD) for all measures considered for the three groups over the four assessment points. No statistical interaction effect (group × phase) was found (Table 2, right) and the main group effect was statistically significant for the SATAQ-R internalisation (*P* = .049) and self-esteem (*P* = .005) scores. When compared to participants in the control group, the adolescents who received the ML + NUT program scored 0.25 points less on SATAQ-R internalisation scores (95% CI [0.06; 0.45]; *P* = .013; *d* = 0.47) and 1.66 points more on self-esteem scores (95% CI [0.54; 2.79]; *P* = .004; *d* = 0.57). When compared to the control group, participants who received the* Theatre Alive* program also scored 1.58 points higher on self-esteem scores (95% CI [0.41; 2.75]; *P* = .008; *d* = 0.50), whereas differences regarding SATAQ-R internalisation scores were only nearly statistically significant (95% CI [−0.00; 0.40]; *P* = .054; *d* = 0.39).  Figure 2 shows profiles over time for the aforementioned findings (adjusted means by preprogram levels and BMI).

## 4. Discussion

This study used a longitudinal design with a follow-up at 5 and 13 months in a school-based ongoing prevention program with a universal mixed-gender sample. The aim was mainly to reduce disturbed eating attitudes and diminish ideal aesthetic internalization through an interactive multimedia program based on media literacy or a program focused on the same topics but based on the dramatic arts, when compared to a control group. The main effect of intervention was statistically significant for some variables, since participants in both experimental groups (ML + NUT and* Theatre Alive* programs) showed significantly higher self-esteem scores than the control group after the intervention and at the 5-month and 13-month follow-ups. Furthermore, the ML + NUT group showed lower aesthetic ideal internalization scores than the control group after the intervention and at later time points. Therefore, these findings suggest both that the two prevention programs are effective in producing significant changes in the treated groups, increasing their self-esteem and partially reducing their internalization, and that these changes are sustained over time.

Ideal internalization has been posited to be an important distal variable leading to ED [48], a precursor of body dissatisfaction [49], and it has been shown to be a causal risk factor in body dissatisfaction [3]. As Thompson's tripartite influence model suggests [50], sociocultural influences play an important role in the process of internalizing cultural ideals of beauty. The acceptance of these unattainable ideals encourages body dissatisfaction and compensatory behaviours to lose weight, increasing the risk for eating disorders [51–53].

Considering the sequence of variables that lead to an ED from a longitudinal viewpoint, it seems that beauty ideal internalization and self-esteem might be distal variables that are at the beginning of the causal sequence. Therefore, it is more likely that in a sample derived from the general population, where we are unlikely to find many individuals with an ED, only a few individuals would show high scores on some of the variables studied, so we cannot expect to get major changes in all the measures. These few individuals are likely to be in the early stages of developing an eating problem. The influence of the programs is mainly exerted on the distal variables, which play a more important role in the beginning of the pathogenic process. In this light, the programs show a protective effect on core psychological variables related to life-skills, which are essential to adaptive adolescent development. Additionally, there is evidence that girls who have developed media literacy skills mitigated the negative influence of the unachievable body image ideal [51]; therefore, we can expect that individuals who have more fully internalized the aesthetic body ideal will experience a greater reduction in this through the multimedia literacy program, although this change was not great enough to be reflected in the body dissatisfaction measure. There is some evidence that short-term media literacy interventions can reduce the internalization of sociocultural ideals; nonetheless, a long-term media literacy intervention is needed to produce changes in body dissatisfaction [54]. Nevertheless, it appears that even preventive interventions specifically targeting body image do not yield significant shifts [49], and when they do occur, the effect sizes are small [45]. One explanation may be that body dissatisfaction is less evident in younger adolescents [55]. Perhaps reactive episodes of body dissatisfaction as a negative consequence of media exposure might accumulate over time, leading to the development of longer-term body dissatisfaction later, as suggested by Hausenblas et al. [7].

In the literature on universal preventive programs targeted at adolescents, it has been found that positive changes in the participants' knowledge level were attained, while favourable changes in attitudes and especially in behaviours were more difficult to attain [56]. In this study, we have achieved an attitudinal change in one of the prevention programs by reducing beauty ideal internalization, but not enough to be reflected in disturbed eating attitudes and body dissatisfaction. According to Piran [56], changes in thinness internalization are more commonly found in this kind of study than changes in body dissatisfaction or disturbed eating attitudes.

The results suggest that the process of body ideal internalization is not the most antecedent variable in pathways toward body dissatisfaction. Likewise, Karazsia et al. [57] uphold that other variables may serve as moderators of the relationship between societal influences promoting aesthetic ideals and body dissatisfaction. Two decades ago, it was suggested that self-esteem might moderate the association between social pressure to be thin and ideal internalization [58]. Thus, individuals with low self-esteem would be more vulnerable to social pressure to be thin and more likely to assume images conveyed in the media as life goals to achieve. Likewise, women with lower self-esteem are especially prone to internalizing social aesthetic ideals in order to be socially accepted [59]. Given the links between internalization and self-esteem, we might expect that interventions which modify self-esteem would be capable of reducing the level of aesthetic ideal adherence, as our outcomes show with the ML + NUT program. According to Cohen's effect size classification, for the ML + NUT program we obtained nearly moderate effects for aesthetical ideal internalization (*d* = 0.47) and moderate self-esteem (*d* = 0.57) when compared to the control group. Working on life-skills variables through media literacy preventive programs could strengthen the participants by making them more resilient to harmful media influences, limiting the impact on body dissatisfaction and interrupting the progress towards dangerous weight loss behaviours and later towards an ED.

Our findings also suggest that the* Theatre Alive* program may be an effective strategy for use in school-based interventions aimed at preventing ED by increasing self-esteem, although its effects may not be important enough to reduce the level of internalization. It appears that the effects of the* Theatre Alive* program are less specific than those of the ML + NUT program but still moderate for self-esteem (*d* = 0.50) when compared to the control group. We believe that preventive drama programs are a promising methodology to involve children and their families with health-related messages and an excellent framework for increased resilience to comments from others. They yield positive changes in communication with them and offer a peer leadership opportunity in which children can teach others. Finally, these learning processes help to boost the participants' self-esteem.

Although our drama-based intervention was inspired by the philosophy of media literacy as well (if we take the content into account), it is not based so much on engaging participants in cognitive criticism and actively challenging these contents as on learning and memorizing a script with health promotion messages and finally performing it. The* Theatre Alive* program could be improved, as suggested by Haines et al. [21], by giving participants an active role in developing the script and giving them the chance to bring their own life experiences to the project. Thus, our drama-based program was useful for increasing the participants' overall self-esteem, as might be expected from a program in which adolescents affirm themselves to a significant audience (parents, teachers, and classmates) and receive public social reinforcement.

Taking these results together, our hypotheses have been partially fulfilled. Both programs can benefit students' self-esteem; moreover, our ML + NUT program was useful in reducing ideal internalization, although the broader hypothesised effects on the other specific risk factors such as reduced body dissatisfaction or ED symptoms were not found. In addition to these reasons, it is possible that the weak internal consistency SCOFF could contribute to the absence of significant changes in eating disorder symptoms and maybe EAT is not the best instrument to detect risk for eating disorders at this subclinical stage as well.

Some limitations should be taken into account when interpreting these results. First, although we used a control group for comparison purposes, this group did not receive any placebo intervention but continued to maintain their regular school activities. Second, we only used self-reported measures, which are very likely to be underreported [9] compared to structured interviews. Third, we are aware that the sampling procedure (by schools) would be vulnerable for the results to some bias involving social factors derived from belonging to the same class group that would increase the effects of the program. Fourth, keeping in mind the role of the parents in shaping beliefs and behaviours regarding the shape, eating, and weight, we recommend extending the preventing program to the parents and to the educative community.

One of the strengths of this study is that it compares two preventive programs which are linked by a common media literacy philosophy in the same study, one of them with an innovative methodology, the* Theatre Alive* program, and the other with an interactive and multimedia format. Furthermore, we believe that another interesting contribution is developing a media literacy program to challenge the masculine and feminine aesthetic models, the manipulations, and cheatings involved in how both models are conveyed in the media targeted at both female and male adolescents. It is necessary to target preventive efforts to the male population [52], and there are few studies in this area. Moreover, as it has been recommended that follow-ups of preventive programs should be done at least once a year [56], another significant strong point of our study was including 5- and 13-month follow-ups. However, the findings with our peer-based interventions which challenge internalization of the thin ideal are promising, since these interventions can protect against the development of eating pathologies [60].

## 5. Conclusions

Our study suggests that the two prevention programs are effective in increasing the adolescent's self-esteem and partially reducing their internalization and that these changes are stable over time. The influence of the programs is mainly exerted on distal variables working in the beginning of the pathogenic process. Both of the programs would show a protective effect on psychological variables deemed as life-skills. Insisting on life-skills variables through media literacy preventive programs could strengthen the participants by making them more resilient to detrimental media influences, reducing the impact on body dissatisfaction and stopping the progress towards an ED.

A great deal of research is needed to clarify these results, and it might be interesting to compare the outcomes of both drama-based methodologies and more conventional media literacy programs targeting ED in larger samples, thereby also increasing their statistical power, with a dismantling approach to determine the beneficial components and the addition of booster sessions and more extensive follow-up to avoid a decrease in benefits from the prevention program [61].

## Figures and Tables

**Table 1:** Eating disorder prevention program contents.

NUT	(A) Definition of balanced eating Definition of and differentiation between nutrition and eating Nutrients Food pyramid and foods Water (B) Analysis of menus: balanced menu menu with high levels of fats menu with high levels of proteins menu with high levels of carbohydrates (C) Final recommendations (NAOS pyramid)	Detailed self-recording of food consumed in the five meals a day for a week “The pleasure of eating”: group activity of testing of the five senses with four different foods (smell, taste, texture, and definition of the food tasted)	

ML	(A) Feminine beauty throughout history: the canon of beauty proposed by the Greeks beauty through art beauty through fashion (B) Recent changes in the criteria for beauty: the Marilyn icon the Barbie icon Drastic changes in sizes for female models (C) Beauty in the world: the deformed feet of Chinese women the “giraffe-women” of Myanmar the extremely obese women of Mauritania the thinness of the West (D) Analysis of advertising messages and transmission of values: what advertising hides from us: real effectiveness of diets and their effects “nothing is as it seems” (example of a nonexistent ideal) manipulation of advertisements (fragmentation, make-up techniques) (E) Economic interests that lie behind the beauty industry Questioning the automatic association between beauty and happiness Real women: a variety of sizes	(A) Masculine beauty throughout history: the canon of beauty proposed by the Greeks beauty through art beauty through fashion (B) Recent changes in the criteria for beauty: historical beauty references of masculinity in the 20th century: Marlon Brando, James Dean, Gregory Peck, and the legendary James Bond evolution in action toys drastic changes in the muscle development of male models (C) Beauty in the world: labial discs of the tribe men Kisêdje crocodile scarification in some tribes of Africa New Guinea Moko Maori Tattoo in New Zealand muscles in the West (D) Analysis of advertising messages and transmission of values: what advertising hides from us: real effectiveness of diets, supplements, and anabolic risks systematic omission of **majority** silhouettes manipulation of advertisements (fragmentation, make-up techniques, PhotoShop, and Moviereshape) (E) Economic interests that lie behind the beauty industry Questioning the automatic association between beauty and happiness Real people: a variety of sizes	Comparison of feminine and masculine aesthetic models Main differences between models: aspirations in relation to your body body areas of concern Means to achieve the perfect body: diet and exercise Possible reasons for these differences: level variation in the aesthetic models throughout history (more feminine) coexistence of different aesthetic ideals in both sexes promotion of roles in male and female toys cultural differences in both aesthetic models globalization of Western aesthetic ideals in both models Manifestation of the models in the media: gender differences, valuing social referents differences in body exposure promotion of traditional gender roles historic changes in gender roles messages that promote the purchase of beauty products aimed at both sexes tricks used (we use the same tricks for both models)

Activism	How to criticise media advertisements How to write a letter of complaint: group discussion Write and send a letter of complaint	How to criticise media advertisements Video script: group discussion Video making-of Videos award	

**Table 2:** Observed means (and SD) of measures over time and ANOVA results.

Measure (minimum ÷ maximum)	Group (*n*)	Observed mean (SD)				ANOVA: *F* (*P* value)		
		Pretest	Posttest	1st follow-up	2nd follow-up	Interaction	Group	Time
		(baseline)	(month 1)	(month 5)	(month 13)			
EAT (0 ÷ 78)	ML + NUT (48)	7.04 (9.85)	5.81 (9.96)	6.10 (8.86)	6.40 (9.13)	0.99 (.376)	1.88 (.156)	0.59 (.442)
	Theatre (43)	7.35 (9.11)	6.23 (8.77)	6.51 (8.88)	5.60 (5.56)			
	Control (64)	5.94 (7.32)	5.78 (7.66)	7.56 (8.39)	6.38 (8.12)			

SCOFF (0 ÷ 5)	ML + NUT (48)	0.83 (1.21)	0.83 (1.26)	0.65 (1.06)	0.60 (0.82)	2.31 (.058)	0.20 (.816)	0.09 (.912)
	Theatre (43)	0.74 (0.98)	0.70 (0.94)	0.72 (0.96)	0.70 (0.94)			
	Control (64)	0.98 (1.08)	0.67 (0.93)	0.94 (1.14)	0.56 (0.99)			

SATAQ-R internalisation (1 ÷ 5)	ML + NUT (47)	2.60 (0.92)	2.40 (0.97)	2.31 (0.96)	2.53 (0.87)	2.36 (.098)	**3.67 (.028)**	0.95 (.332)
	Theatre (43)	2.71 (0.91)	2.58 (0.88)	2.56 (1.03)	2.64 (0.96)			
	Control (63)	2.87 (0.72)	2.76 (0.81)	2.98 (0.78)	2.85 (0.80)			

SATAQ-R awareness (1 ÷ 5)	ML + NUT (47)	3.35 (0.76)	3.30 (0.92)	3.21 (0.88)	3.40 (0.87)	2.09 (.082)	2.56 (.081)	2.55 (.080)
	Theatre (43)	3.58 (0.72)	3.40 (0.74)	3.49 (0.93)	3.50 (0.82)			
	Control (63)	3.63 (0.65)	3.63 (0.72)	3.76 (0.64)	3.59 (0.71)			

CDRS body dissatisfaction (0 ÷ 8)	ML + NUT (49)	0.84 (1.12)	0.76 (0.94)	0.88 (0.83)	0.80 (0.98)	0.62 (.646)	0.62 (.538)	1.89 (.153)
	Theatre (43)	0.87 (1.01)	0.81 (0.90)	0.94 (0.83)	1.02 (1.06)			
	Control (64)	0.84 (0.91)	0.79 (0.68)	0.77 (0.69)	0.86 (0.85)			

RSES self-esteem (10 ÷ 40)	ML + NUT (48)	30.19 (6.02)	31.44 (5.74)	31.90 (6.02)	31.15 (5.20)	2.25 (.109)	**5.59 (.005)**	1.89 (.171)
	Theatre (43)	30.40 (5.12)	30.88 (4.87)	32.21 (5.32)	30.81 (5.28)			
	Control (64)	30.58 (4.02)	30.25 (5.52)	29.59 (4.88)	30.34 (5.39)			

Note: results in bold are significant at .05 level.
